# 中性粒细胞驱动肺腺癌进展的分子机制

**DOI:** 10.3779/j.issn.1009-3419.2025.106.29

**Published:** 2025-10-20

**Authors:** Xiaoya LI, Long LI

**Affiliations:** ^1^730000 兰州，兰州大学第一临床医学院（李小雅）; ^1^The First Clinical Medical School, Lanzhou University; ^2^兰州大学第一医院呼吸与危重症医学科（李龙）; ^2^Department of Respiratory and Critical Care Medicine, The First Hospital of Lanzhou University, Lanzhou 730000, China

**Keywords:** 肺肿瘤, 中性粒细胞, 肿瘤微环境, 中性粒细胞胞外诱捕网, 分子机制, Lung neoplasms, Neutrophils, Tumor microenvironment, Neutrophil extracellular traps, Molecular mechanism

## Abstract

肺癌是全球发病率和死亡率最高的恶性肿瘤之一，肺腺癌（lung adenocarcinoma, LUAD）为主要类型。近年来，研究发现肿瘤微环境（tumor microenvironment, TME）在LUAD的发生发展中扮演重要角色，其中，中性粒细胞的作用日益受到关注。研究发现中性粒细胞在TME中会分化为不同表型，呈现出多重促瘤功能。此外，中性粒细胞在相关刺激下会分泌中性粒细胞胞外诱捕网（neutrophil extracellular traps, NETs），尽管NETs有一定的抗肿瘤特性，但越来越多的研究发现其有多重促肿瘤功能。本综述从免疫调控、细胞代谢、基因及多种分子机制描述了中性粒细胞在LUAD发生发展及转移中的作用。

## Abstract (en)

Lung cancer is one of the most prevalent and lethal malignant tumors worldwide, with lung adenocarcinoma (LUAD) being a major subtype. In recent years, research has revealed that the tumor microenvironment (TME) plays a crucial role in the development and progression of LUAD. Notably, the role of neutrophils has increasingly garnered attention. Studies have shown that neutrophils can differentiate into various phenotypes within the TME, exhibiting multiple pro-tumorigenic functions. Additionally, neutrophils can secrete neutrophil extracellular traps (NETs) in response to certain stimuli. Although NETs possess anti-tumor characteristics, an increasing number of studies have uncovered their multifaceted pro-tumor functions. This review describes the role of neutrophils in the initiation, development, and metastasis of LUAD, focusing on immunoregulation, cellular metabolism, genetics, and various molecular mechanisms.

肺癌具有高度侵袭性和异质性，其突变负荷与组织学分型独特，是癌症相关死亡的首要原因之一^[1]^，其中肺腺癌（lung adenocarcinoma, LUAD）为最常见的类型。随着高通量测序技术的发展，研究者对LUAD的肿瘤微环境（tumor microenvironment, TME）组成和功能获得新认知^[2]^。实体瘤的TME中聚集了复杂的先天性与获得性免疫炎症细胞，中性粒细胞占人类循环白细胞的40%-70%^[3]^。传统观点将其视为先天免疫系统的“机动步兵”，主要负责对病原体的快速反应，然而在TME中，中性粒细胞会分化为肿瘤相关中性粒细胞（tumor-associated neutrophils, TANs），TANs与TME相互作用，通过复杂的机制促进LUAD的发生发展。中性粒细胞胞外诱捕网（neutrophil extracellular traps, NETs）是由活化的中性粒细胞释放的DNA-组蛋白-蛋白质复合体，研究^[4]^证明NETs在肿瘤进展、转移、免疫抑制等过程中发挥关键作用。因此，研究中性粒细胞促进LUAD进展的分子机制，有利于靶向诊治从而改善LUAD患者的预后。

## 1 中性粒细胞分类及其在LUAD中的作用

### 1.1 中性粒细胞的分类

中性粒细胞具有异质性和可塑性，其异质性体现在多个维度：成熟状态、密度、表面标志物和功能极化。中性粒细胞能够根据TME的信号调整其转录程序和功能状态，这种可塑性使其能够在不同的表型和功能状态之间转换^[5]^。TME以慢性炎症为特征，在TME中转化生长因子-β（transforming growth factor-β, TGF-β）导致TANs表型极化，形成N1和N2两种类型^[3]^：N1型TANs抗肿瘤，主要通过直接杀伤肿瘤细胞或激活抗肿瘤免疫来介导肿瘤杀伤；N2型TANs促肿瘤，通过重塑细胞外基质（extracellular matrix, ECM）、加速血管生成和淋巴管生成、介导转移和免疫抑制来促进肿瘤的发展^[6]^。在肿瘤组织中还发现了以促肿瘤和免疫抑制活性为特征的多形核髓源性抑制细胞（polymorphonuclear myeloid-derived suppressor cells, PMN-MDSCs），TME中的中性粒细胞还可依据密度梯度进行分类：成熟（分叶核）高密度中性粒细胞具有抗肿瘤活性，而未成熟（杆状核）低密度中性粒细胞（包括PMN-MDSCs）则表现出与癌症患者循环系统一致的免疫抑制功能^[3]^。一项单细胞RNA测序（single-cell RNA sequencing, scRNA-seq）技术^[7]^对多器官肿瘤组织中的中性粒细胞深度解析后发现，TME中存在T1、T2、T3三种特征性中性粒细胞亚群，T1为未成熟中性粒细胞，T2为成熟中性粒细胞，T1、T2在肿瘤中汇聚成T3，T3表达dcTRAIL-R1，呈现为中间成熟度。T1和T2未表现出明显的促瘤作用，但可转化为具有促瘤功能的T3亚群。一组表面表达唾液酸结合免疫球蛋白样凝集素F（sialic acid-binding immunoglobulin-like lectin F, Siglec-F）的中性粒细胞具有促肿瘤活性^[8]^，且SiglecF^+^中性粒细胞在TME中存活超过6-8 d^[9]^，而正常中性粒细胞生存时间较短。这些结果表明在TME中，中性粒细胞具有异质性和可塑性，且向适应肿瘤生长的方向演变。

### 1.2 中性粒细胞在LUAD中的作用

中性粒细胞通过吞噬作用、形成NETs、产生活性氧（reactive oxygen species, ROS）以及脱颗粒[髓过氧化物酶（myeloperoxidase, MPO）、中性粒细胞弹性蛋白酶（neutrophil elastase, ELANE）、基质金属蛋白酶8（matrix metalloproteinase 8, MMP8）和MMP9]等机制对肿瘤产生直接的细胞毒作用，从而有效抑制肿瘤生长^[10-12]^。然而，在TME的影响下，中性粒细胞可发生表型转化，转变为促瘤表型，进而促进肿瘤血管生成、肿瘤生长和转移^[13]^。此外，研究^[4]^表明，NETs在肿瘤进展、转移和免疫抑制等过程中发挥着关键作用。NETs是由激活的中性粒细胞释放的网状结构，具有抗肿瘤和促肿瘤双重作用^[6]^。当中性粒细胞受到微生物、细胞因子、化学物质、免疫复合物或肿瘤细胞的刺激时，可诱导NETs的形成^[14]^。一方面，NETs中的成分如MPO、组蛋白和蛋白酶可以直接杀伤肿瘤细胞并抑制其生长和转移潜能^[15]^；另一方面，研究^[6]^表明NETs具有促肿瘤效应，NETs可以通过蛋白酶或激活信号直接促进肿瘤细胞增殖，NETs解聚后的三维DNA纤维状网络可以物理捕获循环中的癌细胞，进而促进其扩散和转移，NETs还可以通过蛋白酶降解ECM，从而促进血管内皮生长因子（vascular endothelial growth factor, VEGF）的释放，增强肿瘤侵袭和血管生成^[16]^。此外，NETs通过增加肿瘤细胞中的线粒体生物合成，进而促进肿瘤生长^[6]^。中性粒细胞及NETs胞外诱捕网在TME中的积累可能通过以下机制影响LUAD进展。

#### 1.2.1 免疫调节作用

中性粒细胞通过释放ROS和蛋白酶促进肿瘤细胞侵袭，同时抑制T细胞功能，帮助肿瘤免疫逃逸^[17]^。此外，中性粒细胞可通过铁死亡途径诱导免疫抑制，单细胞转录组分析发现铁死亡途径富集在结直肠癌肝转移中的PMN-MDSCs中^[18]^，PMN-MDSCs在TME中的自发铁死亡触发了前列腺素E2（prostaglandin E2, PGE2）的分泌和氧化磷脂的释放，进而影响CD8^+ ^T细胞和TANs的活性，增强免疫抑制性^[19]^。因此，靶向铁死亡抑制可以作为癌症免疫治疗的治疗策略。在LUAD中PMN-MDSCs容易发生铁死亡，而胰岛素样生长因子结合蛋白1（insulin-like growth factor binding protein 1, *IGFBP1*）的表达与PMN-MDSCs的浸润水平呈正相关，*IGFBP1*高表达表明非小细胞肺癌患者预后不良，这可能与中性粒细胞铁死亡引起的肿瘤免疫抑制有关^[20]^。除铁死亡外，NETs已被证明可以促进肿瘤的免疫逃逸，NETs-DNA结构作为一种物理屏障，可以限制癌细胞与细胞毒性自然杀伤（natural killer, NK）细胞或T细胞的接触^[21]^。此外，NETs结合蛋白还可以诱导免疫抑制，例如，NETs上可以检测到程序性死亡配体-1（programmed death-ligand 1, PD-L1）的表达，这可能导致T细胞耗竭^[22]^。NETs在TME中的免疫抑制作用主要涉及触发CD8^+ ^T细胞的耗竭和功能障碍^[23-24]^、调节Th1和Th2 CD4^+ ^T细胞之间的平衡，并损害NK细胞的抗肿瘤效应^[25]^。

#### 1.2.2 代谢调控

TME中，中性粒细胞不仅表型向促肿瘤方向转变，其代谢模式也发生适应性改变。例如，TANs中葡萄糖转运蛋白1（glucose transporter 1, GLUT1）的上调促进了细胞对葡萄糖的利用增加，这有助于肿瘤生长。癌细胞代谢重编程不仅涉及糖酵解途径的变化，还包括脂肪酸和氨基酸代谢过程的显著改变，这些代谢转变有助于增强细胞内的蛋白质合成和结构修饰，对维持癌细胞的增殖和转移至关重要^[26-28]^。此外，NETs所含的组蛋白、MPO及ROS等组分与脂质过氧化过程密切关联，提示NETs可能通过调控脂质代谢途径来影响肿瘤的生物学行为^[29]^。研究^[9]^还发现，中性粒细胞与HALLMARK_HEME_METABOLISM通路负相关，提示其可能通过干扰血红素代谢影响肿瘤能量供应。

#### 1.2.3 基因调控

通过差异基因分析筛选出脂肪酸结合蛋白7（fatty acid-binding protein 7, *FABP7*）等与中性粒细胞密切相关的基因，基于FABP7表达的风险评分与LUAD患者生存预后显著关联，且FABP7在中性粒细胞高浸润组中差异表达，作为脂质代谢调控因子，FABP7可能参与肿瘤的代谢重编程，进而影响肿瘤进展及耐药性^[30]^。此外，肿瘤坏死因子α诱导蛋白6（tumor necrosis factor alpha-induced protein 6, *TNFAIP6*）作为促肿瘤基因之一，在LUAD细胞中显著过表达，并可能导致中性粒细胞的“N2”型极化^[17]^。

## 2 中性粒细胞促进LUAD进展的多维分子机制

中性粒细胞经趋化因子及细胞因子募集至TME中，其表型可从抗肿瘤N1型极化为促肿瘤N2型，这一过程受多种信号通路调控，TGF-β信号通路是驱动N1型向N2型极化的关键因素。TANs在肿瘤细胞分泌的粒细胞-巨噬细胞集落刺激因子（granulocyte-macrophage colony-stimulating factor, GM-CSF）的诱导下产生抗凋亡蛋白B细胞淋巴瘤超大蛋白（B-cell lymphoma-extra large, BCL-XL），BCL-XL可通过抑制凋亡延长TANs的存活时间，进而有利于TANs与TME相互作用，并通过不同分子机制发挥多重促癌作用，因此靶向这些通路有利于抑制LUAD的生长、侵袭、转移及耐药性，从而提高LUAD患者的预后。

### 2.1 中性粒细胞向TME募集、极化的调控机制

#### 2.1.1 NK2同源盒1（NK2 homeobox 1, NKX2-1）下调激活趋化因子CXCLs/CXCR2轴促进中性粒细胞浸润与LUAD进展

研究^[31]^表明NKX2-1为TME中的关键调节因子，NKX2-1表达降低会激活CXCLs/CXCR2轴，从而促进中性粒细胞的募集和浸润增加。这些中性粒细胞在肿瘤内表现出促癌特性，并具有强烈的细胞间通讯，营造了一个促肿瘤发生的TME，最终推动了LUAD进展。在LUAD中CXCR2与肿瘤侵袭、血管生成和转移有关。此外，CXCLs/CXCR2轴在促进LUAD的耐药性方面也起着至关重要的作用^[32]^。肿瘤细胞表达CXCR，以吸引中性粒细胞到肿瘤生长部位，诱导NETs的形成^[10]^。因此，靶向CXCLs/CXCR2信号通路可能是一种可行的治疗策略，可抑制肿瘤发生并改善NKX2-1低表达LUAD患者的生存结局。

#### 2.1.2 微小RNA-941（microRNA-941, miR-941）/叉头盒N4（fork head box N4, FOXN4）/TGF-β信号轴驱动N2型TANs极化

研究^[33]^表明miRNA可以调控LUAD的肿瘤发生和进展，如miR-941/FOXN4/TGF-β轴驱动N2型TANs极化，从而促进LUAD进展。通过生物信息学分析^[34]^，研究者发现LUAD患者N2型TANs的miR-941活性显著增加，通过预测miR-941下游的潜在靶点，发现仅FOXN4与LUAD患者生存率正相关。此外，经miR-941处理后，细胞中FOXN4蛋白表达水平显著下调。荧光素酶实验显示miR-941模拟物显著降低了*FOXN4* 3'非翻译区野生型（FOXN4 3'-untranslated region wild-type, *FOXN4* 3'-UTR-WT）组的荧光素酶活性，而在*FOXN4* 3'非翻译区突变（*FOXN4* 3'-untranslated region mutant, *FOXN4* 3'-UTR-MUT）组中未观察到显著变化，这支持了miR-941抑制LUAD中FOXN4表达的结论。FOXN4已被报道可抑制癌症进展^[35]^，研究^[34]^显示多个促进肿瘤进展的通路在FOXN4高表达组中被抑制，包括TGF-β信号通路、缺氧、上皮间质转化（epithelial-mesenchymal transition, EMT）、糖酵解、血管生成和K-RAS信号通路，这些结果揭示FOXN4在LUAD中通过抑制这些通路发挥其肿瘤抑制功能。综合上述分析，研究者揭示了一条关键的调控轴：N2型TANs的miR-941↑→FOXN4↓→TGF-β↑→诱导更多N1型向N2型转化→miR-941活性增强进一步放大环路，通过靶向抑制miR-941或联合TGF-β阻断剂干预该轴，能够有效逆转免疫抑制性TME。此外，外周血miR-941水平可作为LUAD预后或免疫治疗反应的预测指标。

#### 2.1.3 内皮黏蛋白（endomucin, EMCN）缺失通过诱导中性粒细胞招募与N2型极化，驱动肿瘤肺转移

中性粒细胞在肿瘤肺转移的转移前微环境形成中发挥关键作用^[36]^。研究人员通过核糖核酸测序（RNA sequencing, RNA-seq）和免疫荧光染色分析了野生型（wild type, WT）和EMCN^ecKO^（EMCN特异性敲除）肿瘤荷瘤小鼠的转移前肺部微环境，发现与WT肿瘤荷瘤小鼠相比，EMCN^ecKO^肿瘤荷瘤小鼠的肺部显著招募了Ly6G^+^和S100A9^+^中性粒细胞，表明EMCN缺失可以在肺部引起中性粒细胞浸润。进一步的实验表明，通过抗Ly6G抗体耗竭中性粒细胞后，EMCN^ecKO^小鼠的肺转移灶明显减少。以上结果表明，Ly6G^+^中性粒细胞在由EMCN缺失引起的肿瘤肺转移中发挥了重要作用。TGF-β在诱导N1型向N2型中性粒细胞极化中起着重要作用，通过癌症基因组图谱（The Cancer Genome Atlas, TCGA）数据集的生物信息学分析和实验验证^[37]^，研究者发现EMCN与TGF-β存在负相关，因此，研究者假设EMCN缺失导致微环境中TGF-β增加，促进了N1型中性粒细胞向N2型极化，从而促进了肺转移。为验证EMCN缺失通过TGF-β介导N2型极化，将路易斯肺癌（Lewis lung carcinoma, LLC）细胞皮下注射到WT和EMCN^ecKO^小鼠体内，研究者对EMCN^ecKO^小鼠进行抗TGF-β抗体治疗或不治疗，并检测N1型、N2型中性粒细胞标志物，结果显示EMCN缺失促进了原发肿瘤的肺转移，抗TGF-β抗体抑制了由EMCN缺失引起的肺转移。免疫荧光分析进一步证实，EMCN^ecKO^小鼠肺转移灶中N2型中性粒细胞标志物表达升高，而抗TGF-β抗体治疗后N1型中性粒细胞标志物表达增加。这些结果表明EMCN缺失通过TGF-β依赖性机制驱动中性粒细胞招募及N2型极化，促进肺转移。靶向TGF-β可逆转中性粒细胞极化，为肺转移防治提供新策略。

### 2.2 TANs多重促癌机制

#### 2.2.1 GM-CSF介导的Janus激酶（Janus kinase, JAK）/信号转导与转录激活因子（signal transducer and activators of transcription, STAT）/BCL-XL信号通路促进中性粒细胞存活与促瘤功能

在LUAD基因工程小鼠模型中，肿瘤细胞通过GM-CSF介导的JAK/STAT信号通路诱导中性粒细胞表达抗凋亡蛋白BCL-XL^[38]^，BCL-XL具有抗凋亡作用，可阻断TANs的凋亡过程，进一步研究发现，BCL-XL与肿瘤细胞增殖和化疗耐药性相关。对中性粒细胞亚群进行分析，结果显示SiglecF^+^中性粒细胞亚群具有促肿瘤活性^[8]^，有研究^[9]^发现SiglecF^+^中性粒细胞在肺TME中可以存活超过6-8 d。肿瘤细胞局部分泌GM-CSF可在体内介导TANs向SiglecF^+^、长寿促瘤表型极化。在肾纤维化小鼠模型中GM-CSF可诱导SiglecF^+^中性粒细胞的产生^[39]^。这些研究表明，GM-CSF是促进中性粒细胞存活并使其向支持肿瘤的细胞表型转变的关键因素。

#### 2.2.2 原肌球蛋白2（tropomyosin 2, TPM2）下调通过ELANE激活促进肿瘤进展

TPM2在调节细胞增殖、迁移、凋亡、囊泡运输和细胞分裂中发挥重要作用，有研究^[40]^报道，TPM2在LUAD组织中表达下调，且与LUAD患者的不良预后有关。ELANE是中性粒细胞初级颗粒中的主要蛋白，其在LUAD患者中的活性显著高于慢性阻塞性肺疾病、肺纤维化等。通过实验发现，外源性TPM2能以正向剂量依赖的方式下调中性粒细胞中ELANE和p38丝裂原活化蛋白激酶（p38 mitogen-activated protein kinase, p38 MAPK）的表达水平，使用p38 MAPK通路特异性抑制剂SB202190处理后，由TPM2下调引起的ELANE表达升高被逆转。以上实验证明，LUAD肿瘤细胞中TPM2下调，通过p38 MAPK信号通路的激活增加中性粒细胞中的ELANE水平。而ELANE通过Hippo信号通路促进肿瘤细胞的进展，Hippo信号通路是器官大小、组织稳态和肿瘤发生的关键调节因子，在Hippo信号通路下游，Yes相关蛋白（Yes associated protein, YAP）/PDZ结合域的转录共刺激因子（transcriptional coactivator with PDZ-binding motif, TAZ）主要调控与细胞增殖、分化和存活相关靶基因富含半胱氨酸血管生成诱导因子61（cysteine-rich angiogenic inducer 61, CYR61）和结缔组织生长因子（connective tissue growth factor, CTGF）的表达，进而影响器官的生长和肿瘤发生。此外，TPM2的下调激活中性粒细胞中的ELANE，从而促进细胞外信号调控蛋白激酶1/2（extracellular signal-regulated kinase, ERK1/2）的激活，进而增强白细胞介素1β（interleukin-1 beta, IL-1β）和IL-8的分泌，吸引更多中性粒细胞进入TME，影响肿瘤的进展^[41]^。本研究揭示了一条关键的促肿瘤信号轴：TPM2下调→p38 MAPK通路激活→ELANE表达上调→Hippo信号通路活化→促进肿瘤进展。靶向该轴，如开发TPM2激动剂或采用p38 MAPK/ELANE多靶点联合阻断策略，有望为LUAD患者的精准治疗策略提供重要的理论依据和潜在的药物靶点。

#### 2.2.3 G蛋白亚基γ转导蛋白1（G protein subunit gamma transducin 1, *GNGT1*）/纤维蛋白原β链（fibrinogen β chain, *FGB*）/NETs轴重塑TME驱动免疫逃逸与肿瘤进展

通过TCGA和基因表达综合数据库（Gene Expression Omnibus, GEO）分析发现*GNGT1*在LUAD患者的肿瘤组织中表达上调，生存分析显示*GNGT1*高表达会降低患者的总生存期，且*GNGT1*高表达与淋巴结转移、胸膜侵犯和低分化显著关联。LUAD中*GNGT1*的上调表达与基因改变和甲基化的调控网络有关。基于TCGA数据库按*GNGT1*表达水平分组，利用STRING数据库构建蛋白质-蛋白质相互作用（protein-protein interaction, PPI）网络，筛选出*FGB*、白蛋白（albumin, *ALB*）、癌/睾丸抗原2（cancer/testis antigen 2, *CTAG2*）、甲胎蛋白（alpha-fetoprotein, *AFP*）、结合珠蛋白（haptoglobin, *HP*）和成纤维细胞生长因子4（fibroblast growth factor 4, *FGF4*）为枢纽基因，其中*FGB*、*CTAG2*、*AFP*、*HP*与*GNGT1*显著关联。通过在*GNGT1*^fl^*^/+^*和野生型小鼠中检测枢纽基因的mRNA表达水平，并分析与多个生物学过程相关的差异表达基因（differentially expressed genes, DEGs），最终发现*FGB*在所有检测基因中的变化最为显著，表明*FGB*与*GNGT1*存在显著相关性，京都基因和基因组百科全书（Kyoto Encyclopedia of Genes and Genomes, KEGG）通路分析表明FGB通过NETs促进肿瘤进展，在*GNGT1*^fl/+^小鼠模型中，通过实时荧光定量逆转录聚合酶链反应（real-time quantitative reverse transcription polymerase chain reaction, RT-qPCR）验证了NETs形成的关键分子表达显著上调。这些结果表明，肿瘤细胞中GNGT1↑→FGB↑→NETs激活→诱导肿瘤细胞进展及转移^[42]^。

#### 2.2.4 中性粒细胞通过代谢重编程促进LUAD进展

##### 2.2.4.1 NETs通过代谢重编程诱导铁死亡抵抗和抑制CD8^+ ^T细胞的功能

NETs由中性粒细胞生成，NETs通过介导N6-甲基腺苷（N6-methyladenosine, m6A）修饰的溶质载体家族2成员3（solute carrier family 2 member 3 mRNA, SLC2A3）mRNA的稳定性诱导铁死亡抵抗和抑制CD8^+ ^T细胞^[43]^，从而促进LUAD的生长。在LCC与NETs共培养实验中，YTH N6-甲基腺嘌呤RNA结合蛋白F2（YTH N6-methyladenosine RNA binding protein F2, YTHDF2）的表达显著增加，表明NETs上调YTHDF2表达，YTHDF2特异性识别SLC2A3 mRNA的m6A位点，招募CCR4-NOT降解复合体，加速SLC2A3 mRNA降解。*SLC2A3*是GLUT3的编码基因，其降解导致葡萄糖摄取减少→糖酵解抑制→胞内ROS水平降低，ROS水平降低会增强谷胱甘肽过氧化物酶4（glutathione peroxidase 4, GPX4）和溶质载体家族7成员11（solute carrier family 7 member 11, SLC7A11）的活性，从而维持谷胱甘肽（glutathione, GSH）水平，抑制脂质过氧化和铁死亡，最终促进肿瘤增殖与迁移。以上结果表明NETs→YTHDF2↑→YTHDF2识别并结合m6A修饰的SLC2A3 mRNA→SLC2A3 mRNA降解→GPX4、SLC7A11活性↑→铁死亡抵抗，进而促进LUAD发展；CD8^+ ^T细胞分泌的γ干扰素（interferon gamma, IFNγ）激活STAT1信号通路，激活的STAT1信号通路抑制SLC7A11的表达，通过抑制SLC7A11和增加脂质ROS，CD8^+ ^T细胞诱导肿瘤细胞发生铁死亡。佛波酯-12-十四酸酯-13-乙酸酯（phorbol 12-myristate 13-acetate, PMA）可显著诱导NETs形成，该效应可被脱氧核糖核酸酶I（deoxyribonuclease I, DNase I）逆转。PMA处理会减少CD8^+ ^T细胞数量并抑制其细胞毒性，而DNase I能阻断此抑制作用。由于DNase I仅降解DNA且NETs含DNA组分，表明PMA通过NETs抑制CD8^+ ^T细胞功能。NETs通过抑制CD8^+ ^T细胞的功能活性，从而减少IFNγ和肿瘤坏死因子α（tumor necrosis factor alpha, TNF-α）的分泌，上调GPX4、SLC7A11和铁蛋白重链1（ferritin heavy polypeptide 1, FTH1）的表达，下调酰基辅酶A合成酶4（acyl-CoA synthetase 4, ACSL4）和前列腺素内过氧化物合酶2（prostaglandin-endoperoxide synthase 2, PTGS2）的表达，最终保护肿瘤细胞免于铁死亡。NETs可降低脂质ROS和丙二醛（malondialdehyde, MDA）水平，升高GSH水平，进一步抑制铁死亡。此外，NETs可抑制CD8^+ ^T细胞中穿孔素、颗粒酶A和颗粒酶B的表达，减弱其对肿瘤细胞的直接杀伤作用。

##### 2.2.4.2 GLUT1介导的葡萄糖代谢在TANs中的促癌作用及治疗潜力

TANs中GLUT1的高表达显著促进肺癌生长并诱导放疗抵抗^[9]^。在LUAD小鼠模型中，TANs的GLUT1表达和葡萄糖代谢均有所增加。差异表达分析和通路富集分析显示，与健康中性粒细胞相比，TANs中糖酵解通路激活最为显著，表现为GLUT1依赖性葡萄糖摄取增加和ATP生成增强。肺肿瘤产生的可溶性分子通过上调GLUT1增加中性粒细胞存活率，特异性敲除中性粒细胞的GLUT1，诱导多个与中性粒细胞招募相关的基因表达上调、TANs活性下降，从而导致中性粒细胞更新加快，此外，GLUT1缺失会减少促肿瘤亚群SiglecF^+ ^TANs形成、抑制肿瘤生长、增强放疗的敏感性。以上表明TME通过GLUT1依赖性葡萄糖代谢重塑TANs，促进其存活和促肿瘤功能。年轻的中性粒细胞高表达GLUT3，表现为抗肿瘤特性，其在肿瘤中会分化为衰老中性粒细胞，高表达GLUT1和SiglecF，表现为促肿瘤特性，且抑制年轻中性粒细胞招募，从而形成促肿瘤循环。将葡萄糖代谢，尤其是由GLUT1/3介导的葡萄糖摄取，定位为TANs更新的关键节点，该节点可能控制肺癌中促肿瘤中性粒细胞和抗肿瘤中性粒细胞之间的平衡。

##### 2.2.4.3 中性粒细胞脱颗粒产物通过脂代谢重塑肺癌细胞代谢表型

研究^[44]^表明，与基础培养基（basal medium, BM）相比，H1299肺癌细胞（富含脂滴）在含有中性粒细胞脱颗粒产物（neutrophil degranulation products, NDP）的培养基中表现出明显的形态变化和黏附性丧失，部分细胞从培养板脱落，但仍保持活力和增殖特性，而在体内这可能会促进中性粒细胞脱颗粒介导的肺癌细胞向不同组织转移，而H1563细胞（无脂滴）的形态和黏附性不受NDP影响，这表明NDP可能通过脂滴改变细胞黏附性，促进肿瘤细胞的迁移或转移。研究人员用DESeq2 Bioconductor软件包，筛选了与细胞系间NDM/BM差异有关的DEGs，检测到674个与H1299和H1563细胞系间培养基效应差异有关的DEGs，其中许多基因涉及甾醇和胆固醇生物合成过程的调控，这表明中性粒细胞脱颗粒可能通过调控肿瘤细胞代谢相关基因，重塑肿瘤细胞的代谢表型。此外，中性粒细胞会摄取脂质并同时增强脂质合成，这可能导致脂滴的积累^[45]^，在癌细胞增殖期间脂滴可以提供能量储备和细胞膜所需的成分，从而维持细胞存活。

## 3 临床意义及未来展望

中性粒细胞通过多维度调控网络驱动LUAD进展，其机制涵盖：向TME的定向募集、功能表型极化及多重信号调控轴。这些发现为靶向中性粒细胞的治疗策略提供了理论基础，基于上述机制，靶向中性粒细胞的治疗策略可聚焦以下两个核心环节：（1）针对募集过程及功能极化过程，可应用CXCR2抑制剂阻断NKX2-1下调诱导的中性粒细胞募集；静脉输注重组EMCN蛋白恢复内皮屏障完整性，可抑制中性粒细胞浸润与TGF-β释放，进而阻遏N2型TANs极化。TGF-β在TANs表型转化中发挥核心作用，使用抗TGF-β抗体或小分子抑制剂阻断TGF-β信号通路，抑制N1型向N2型极化；（2）针对多维调控机制，靶向治疗实现有效阻断。A-1331852是一种口服强效且高度选择性的BCL-XL阻断剂，其可逆转TANs的老化，增加浸润性T淋巴细胞的比例，且可以抑制肺癌生长，可有效阻断GM-CSF介导的JAK/STAT信号通路。TPM2/p38 MAPK/ELANE/Hippo信号轴构成LUAD进展的关键调控网络，其异常激活通过促进驱动肿瘤细胞增殖及中性粒细胞浸润，显著影响患者预后，该轴的多环节靶向干预策略，包括恢复TPM2表达、抑制p38 MAPK及ELANE活性、阻断Hippo通路，为改善LUAD预后提供了潜在治疗方向。针对GNGT1-FGB-NETs轴在LUAD中的促瘤机制，可通过多环节靶向干预阻断该信号轴的激活，如设计GNGT1的siRNA或shRNA，通过脂质体或病毒载体递送至肿瘤细胞，特异性降解GNGT1 mRNA，抑制其表达，或开发针对FGB蛋白的特异性单克隆抗体或模拟物，阻断或竞争与中性粒细胞表面受体的结合，抑制FGB诱导的NETs形成。YTHDF2是NETs诱导铁死亡抵抗的关键分子，开发针对YTHDF2的小分子抑制剂或siRNA，以阻断其表达和功能。

综上所述，中性粒细胞通过募集、极化及多维信号网络驱动LUAD进展，针对CXCR2、TGF-β、BCL-XL等关键节点的干预策略已展现出治疗潜力。然而，临床转化仍需解决药物递送效率、微环境异质性及免疫平衡等挑战。未来研究应结合单细胞技术与人工智能，开发精准分型工具；通过类器官模型与临床试验的联动，优化联合治疗方案。最终，基于中性粒细胞调控网络的靶向治疗有望成为LUAD综合管理和个性化治疗的重要支柱。
